# Effects of Individualized Follow-Up With an App Postcardiac Rehabilitation: Five-Year Follow-Up of a Randomized Controlled Trial

**DOI:** 10.2196/60256

**Published:** 2025-02-13

**Authors:** Pernille Lunde, Asta Bye, Jostein Grimsmo, Are Hugo Pripp, Vibeke Ritschel, Even Jarstad, Birgitta Blakstad Nilsson

**Affiliations:** 1 Department of Rehabilitation Science and Health Technology Faculty of Health Sciences OsloMet – Oslo Metropolitan University Oslo Norway; 2 Department of Nursing and Health Promotion Faculty of Health Sciences OsloMet – Oslo Metropolitan University Oslo Norway; 3 European Palliative Care Research Centre (PRC) Department of Oncology Oslo University Hospital and Institute of Clinical Medicine, University of Oslo Oslo Norway; 4 Department of Cardiac and Pulmonary Rehabilitation Lovisenberg Rehabilitation Cathinka Guldberg’s Hospital Jessheim Norway; 5 Faculty of Health Sciences OsloMet – Oslo Metropolitan University Oslo Norway; 6 Oslo Centre of Biostatistics and Epidemiology Oslo University Hospital Oslo Norway; 7 Department of Cardiology and Exercise Physiology Norwegian Sport Medicine Clinic (Nimi, part of Volvat) Oslo Norway; 8 Section for Physiotherapy Division of Medicine Oslo University Hospital Oslo Norway

**Keywords:** mHealth, cardiac rehabilitation, mobile phone app, smartphone, lifestyle

## Abstract

**Background:**

Adherence to healthy behaviors initiated or adapted during cardiac rehabilitation (CR) remains a significant challenge, with few patients meeting guideline standards for secondary prevention. The use of mobile health (mHealth) interventions has been proposed as a potential solution to improve adherence to healthy behaviors after CR. In particular, app-based interventions have shown promise due to their ability to provide monitoring and feedback anytime and anywhere. Growing evidence supports the use of apps in post-CR settings to enhance adherence. In 2020, we demonstrated that individualized follow-up via an app increased adherence to healthy behaviors 1 year after CR. However, it remains uncertain whether these effects persist once the follow-up is discontinued.

**Objective:**

This study aims to evaluate the long-term effects of individualized follow-up using an app, assessed 4 years after the intervention.

**Methods:**

A single-blinded multicenter randomized controlled trial was conducted. Patients were recruited from 2 CR centers in eastern Norway. The intervention group (IG) received individualized follow-up through an app for 1 year, while the control group (CG) received usual care. After the 1-year follow-up, the app-based follow-up was discontinued for the IG, and both groups were encouraged to maintain or improve their healthy behaviors based on their individual risk profiles. The primary outcome was the difference in peak oxygen uptake (VO_2peak_). The secondary outcomes included exercise performance, body weight, blood pressure, lipid profile, exercise habits, health-related quality of life, health status, cardiac events, and physical activity. Linear mixed models for repeated measurements were used to analyze differences between groups. All tests were 2-sided, and *P* values ≤0.05 were considered statistically significant.

**Results:**

At the 5-year follow-up, 101 out of the initial 113 randomized participants were reassessed. Intention-to-treat analyses, using a mixed model for repeated measurements, revealed a statistically significant difference (*P*=.04) in exercise habits in favor of the IG, with a mean difference of 0.67 (95% CI 0.04-1.29) exercise sessions per week. Statistically significant differences were also observed in triglycerides (mean difference 0.40, 95% CI 0.00-0.79 mmol/l, *P*=.048) and walking (*P*=.03), but these were in favor of the CG. No differences were found between the groups for other evaluated outcomes.

**Conclusions:**

Most of the benefits derived from the app-based follow-up diminished by 4 years after the intervention. Although the IG reported statistically significantly higher levels of exercise, this did not translate into improved VO_2peak_ or exercise performance. Our study highlights the need for follow-up from health care providers to enhance adherence to healthy behaviors in the long term following CR.

**Trial Registration:**

ClinicalTrials.gov NCT03174106; https://clinicaltrials.gov/ct2/show/NCT03174106 (original study protocol) and NCT05697120; https://clinicaltrials.gov/ct2/show/NCT05697120 (updated study protocol)

## Introduction

The beneficial effects of comprehensive cardiac rehabilitation (CR) have been well established, and for the past decade, CR has carried a class IA recommendation in the European guidelines on cardiovascular disease [1,2]. The overarching goal of CR and secondary prevention is to prevent subsequent cardiac events [1]. Achieving this goal relies heavily on adherence to healthy behavior [1,3]. However, maintaining healthy behavior initiated or adapted during CR is challenging for most patients, resulting in only a small proportion achieving the guideline standards for secondary prevention [3-5]. Accordingly, the long-term (≥1 year) outcomes of CR are poor, and evaluations of CR with follow-ups exceeding 1 year are sparse.

The recently published statement from the European Association of Preventive Cardiology on optimizing adherence in the secondary prevention of cardiovascular disease [3] recommends mobile health (mHealth) interventions to address challenges related to adherence to healthy behavior after CR. mHealth was defined by the World Health Organization in 2011 as “medical and public health practice supported by mobile devices, such as mobile phones, patient monitoring devices, personal digital assistants, and other wireless devices” [6]. It includes the use of mobile phones for calls, short messaging, and more advanced functionalities, such as apps that utilize packet radio service, 3G and 4G systems, global positioning systems, and Bluetooth technology for medical and public health practice [6]. The use of apps was suggested early as particularly promising, as it enables monitoring and providing feedback to patients from anywhere, at any time [7]. Despite rapid advancements in the mHealth research field, only a few studies have investigated whether app use can support adherence to lifestyle changes after completing CR. Duscha and colleagues [8] conducted a randomized controlled trial to evaluate the impact of an mHealth intervention, including an app, on exercise capacity (peak oxygen uptake [VO_2peak_]) after CR [8]. Among 32 patients randomized, they observed a significant increase in absolute VO_2peak_ at the 12-week follow-up. The TeleCare study (n=122) investigated the effects of telemonitoring (smartphone and Bluetooth heart rate monitor belt) and telecoaching (monthly calls over 6 months) compared with usual care on VO_2peak_ and other cardiovascular risk factors [9]. In addition to assessing patients at the 6-month follow-up at the end of the intervention, they re-evaluated them 6 months after the intervention (12 months after CR) [9]. No significant differences were observed between the groups at either follow-up [9].

In 2020, we demonstrated that individualized follow-up for 1 year using an app improved adherence to healthy behaviors after CR [10]. In this study, 113 patients were randomly assigned to individualized follow-up enabled by an app or to a control group (CG) receiving usual care. At the 1-year follow-up, there was a statistically significant difference between the groups, favoring the intervention group (IG), in VO_2peak_ (ml/kg/minute), exercise performance, exercise habits, and self-perceived goal achievement [10]. At the 1-year follow-up, the individualized follow-up ceased, and both the CG and the IG were encouraged to maintain or improve their healthy behaviors based on their individual risk profiles. To the best of the authors’ knowledge, our study was the first to evaluate the effect of an app over an entire year in a post-CR setting. However, no research to date has investigated the long-term effects of successful mHealth interventions beyond 1 year after app use is discontinued. Therefore, the primary aim of this study was to examine whether the improvement in VO_2peak_ observed after 1 year [10] was maintained 4 years after the intervention. Similarly, as secondary outcomes, we aimed to evaluate exercise performance, body weight, resting blood pressure, lipid profile, exercise habits, health-related quality of life (HRQL), health status, cardiac events, and level of physical activity.

## Methods

### Design

We conducted a single-blinded, randomized controlled trial with 2 arms. Patients were allocated to either the CG or the IG in a 1:1 ratio using a computer-generated, permuted block randomization scheme. The randomization was stratified by the CR program. A statistician (AHP) performed the randomization and sent the schemes to one of the coauthors (BBN), who prepared sealed envelopes. The first author, who also recruited the patients and conducted baseline assessments, brought the sealed envelopes to the baseline assessments. The envelopes were opened immediately after each baseline assessment, and patients were informed of their group allocation. The IG received individualized follow-up through an app for 1 year, while the CG received usual care, which included recommendations to maintain or further improve their lifestyle. Reporting adhered to the CONSORT (Consolidated Standards of Reporting Trials) 2010 statement [11].

### Setting and Participants

Patients were recruited from 2 CR centers in the eastern part of Norway. One center offers 1- and 4-week inpatient CR programs, while the other provides a 12-week outpatient CR program. The 12-week outpatient rehabilitation program includes exercise and education sessions 3 times a week, along with personalized support based on patients’ needs, such as medication optimization, psychological counseling, dietary advice, and assistance with smoking cessation. This program has previously been evaluated and shown to improve exercise capacity and quality of life [12]. The 1- and 4-week residential programs had the same content as the 12-week outpatient program. However, the 4-week residential program provided patients with more time to adapt to lifestyle changes and learn about their condition compared with the 1-week program. The 4-week residential program has previously been reported as standard care [13]. Cardiologists, physiotherapists, dietitians, psychologists, and nurses were part of the health care team in all CR programs. Patients were recruited from 3 different CR programs, with randomization stratified accordingly. Approximately one-third of the patients were recruited from each program. Patients attending these CR programs are referred by a physician for various forms of heart disease, with coronary artery disease (CAD) being the most common reason for referral.

During participation in CR, all patients received oral information about the study from the first author (PL). Patients who met the inclusion criteria and were willing and able to participate were scheduled for a baseline assessment. Baseline assessments were conducted after a cardiopulmonary exercise test (CPET), during which patients were screened for exclusion criteria. As previously described [10,14], inclusion criteria included patients completing one of the CR programs, aged ≥40 years, ownership and use of an Android (Google LLC/Alphabet Inc.) or Apple (Apple Inc.) smartphone, and the ability to read and understand Norwegian or English. Exclusion criteria included ischemia or arrhythmias identified during CPET that imposed restrictions equivalent to <80% of maximal heart rate or a Borg scale (6-20) score [15] <15 during exercise. Patients with muscular or skeletal disorders that significantly affected exercise capacity more than their heart disease were excluded. Additionally, patients with severe malignant diseases (ie, advanced cancer) that had a greater impact on life expectancy than their heart disease were also excluded.

Recruitment for the 5-year follow-up was conducted by sending email and SMS text messages to all patients included in the original study [10]. The mail included written information about this study and an appointment for the 5-year follow-up, sent 4-6 weeks before the scheduled appointment. Patients were encouraged to confirm their willingness to participate and notify the first author if they planned to attend. Efforts were made to accommodate each patient’s schedule.

### Intervention Group

After the baseline assessment, patients randomly assigned to the IG were given access to an app and trained on how to use it. The app allowed users to create and set goals, with tasks and automatic reminders. A detailed description of the intervention is provided elsewhere [10,14]. Following the baseline assessment, the app was personalized with each patient’s goals and tasks for the upcoming year. Patients could choose when and how often they wanted reminders for their tasks. Additionally, they could use the app for reflections or notes related to a specific goal and directly communicate any questions to the supervisor. The supervisor had access to an administrator interface, which allowed them to monitor each patient. The interface also enabled the supervisor to send short motivational messages (maximum 112 characters) directly to each patient through the app.

Patients in the IG received comprehensive individualized feedback based on their goals, completed tasks, pending tasks, and any notes they had written. This feedback was provided weekly for the first 12 weeks and monthly for the remainder of the year. Additionally, all patients received 1-3 short individualized motivational messages per week throughout the year. The supervisor, a specialized physiotherapist in this study, provided all feedback and motivational messages to the patients in the IG. Questions submitted to the supervisor were answered within 2 working days. This individualized follow-up continued until the 1-year follow-up assessment. After the 1-year follow-up, the app was removed from the patients’ smartphones, and the follow-up was discontinued. At this point, patients were encouraged to maintain or improve their healthy behaviors based on their individual risk profiles.

### Control Group

Patients allocated to the CG received usual care, which included visits to their general practitioner and cardiologist as needed. After the baseline assessment, they were encouraged to maintain or improve healthy behaviors based on their individual needs and goals. The same encouragement was provided at the 1-year follow-up assessment.

### Outcomes and Assessments

#### Assessment Protocol and Outcome Measures Over a 5-Year Follow-Up Period

Assessments were performed at baseline (post-CR), after 1 year, and after 5 years at the same CR center where the patient was recruited. During the baseline assessment, demographic data were collected. The primary outcome was VO_2peak_. Secondary outcomes at the 1- and 5-year follow-ups included exercise performance (time to exhaustion, peak incline, and peak velocity), body weight, resting blood pressure, lipid profile, exercise habits, HRQL, and health status. Additionally, at the 5-year follow-up, new cardiac events and levels of physical activity were assessed. Test personnel measuring the primary outcome were blinded to group allocation. Data collection was organized and conducted by the same person at all follow-ups, and the same test personnel led the tests at all 3 assessments.

#### Peak Oxygen Uptake and Exercise Performance

To ensure eligibility for the study, all patients performed a CPET before entering the study and at all follow-up times (1 and 5 years). The test was used to determine VO_2peak_ and exercise performance and is described in detail elsewhere [10,14]. Briefly, 2 standardized protocols were created: a walking protocol and a running protocol. Experienced test personnel selected the most suitable protocol for each patient, and the same protocol was used for all 3 tests. During all tests, patients were strongly encouraged to exercise to exhaustion; the Borg scale (6–20) [15] was used to assess perceived exertion, and VO_2peak_ was taken as the highest 30-second VO_2_ measurement. In case of suspected submaximal effort, the test was repeated. Exercise performance was evaluated by time to exhaustion (seconds), peak incline (%), and peak velocity (km/hour). All CPETs were performed with continuous 12-lead electrocardiogram monitoring. Pulmonary ventilation and gases (oxygen and carbon dioxide) were recorded breath-by-breath using a Vyntus CPX metabolic analyzer (Vyaire Medical) at 1 of the CR centers. At the other CR center, pulmonary ventilation and gases were analyzed using a Schiller Ganshorn ergo-spirometry system (Schiller AB) at baseline, and with a Vyntus CPX (Customed) at the 1- and 5-year follow-ups. Patients were instructed to take medication and eat and drink as usual before all tests.

#### Body Weight

Body weight was measured without shoes and while wearing exercise clothes before the CPET at all assessments. Efforts were made to use the same equipment for all measurements.

#### Blood Pressure

Blood pressure was measured manually before the CPET. Patients were instructed to relax in a chair for a minimum of 3-5 minutes before the measurements were taken. Three measurements were performed, preferably on the left arm, and the lowest recorded value was used.

#### Lipid Profile

Venous blood was drawn using standard local procedures at the general practitioner’s clinic within 4 weeks before each assessment. Patients were instructed to fast overnight before providing blood samples. Data were collected on low-density lipoprotein (LDL)-cholesterol, high-density lipoprotein (HDL)-cholesterol, total cholesterol, and triglycerides. Patients were required to bring the results to the assessment points.

#### Exercise Habits

At all assessments, patients were asked about their exercise habits. Exercise habits were defined as the average number of exercise sessions per week over the past year. An exercise session was further defined as a structured activity lasting at least 30 minutes, during which the individual became both sweaty and breathless and felt the need to shower afterward.

#### Health-Related Quality of Life

HRQL was assessed using the HeartQoL questionnaire, a disease-specific HRQL tool comprising 14 questions [16]. This questionnaire is both valid and reliable in patients referred to CR [17-21]. It includes 2 subscales: a physical HRQL subscale (10 items) and an emotional HRQL subscale (4 items) [16]. The combination of these subscales provides a global scale score [16]. Scores range from 0 to 3, with higher scores indicating better HRQL.

#### Health Status

Health status was assessed using the EQ-5D questionnaire, which consists of 5 questions [22]. Each question addresses a different dimension of health status: mobility, self-care, usual activities, pain/discomfort, and anxiety/depression [22]. Each question offers 5 response options, with a score of 1 indicating the best possible outcome and a score of 5 indicating the worst [22]. The EQ-5D also includes an overall health question (EQ Visual Analog Scale), which is answered on a Likert scale ranging from 0 to 100, where 0 represents the worst possible health and 100 represents the best possible health [22].

#### Cardiac Events

At the 5-year follow-up, new cardiac events were assessed. Patients were asked if they had experienced any cardiac events, with follow-up questions posed as needed. New cardiac events were defined as documented acute coronary syndrome, newly diagnosed CAD, valve surgery, survival of cardiac arrest, or atrial fibrillation (AF). Additionally, it was noted whether patients had undergone coronary angiography, percutaneous coronary intervention, or coronary artery bypass grafting, as well as any other cardiac-related incidents.

#### Physical Activity

At the 5-year follow-up, physical activity was assessed using the short version of the International Physical Activity Questionnaire (IPAQ). This questionnaire was designed as a tool for cross-national assessment of physical activity [23]. Both long and short versions of the IPAQ are available, and 2 reference periods can be evaluated: the “last 7 days” or a “usual week” [23]. In this study, the “last 7 days” reference period was used. The short version of the IPAQ provides information on time spent walking, as well as engaging in moderate-intensity and vigorous-intensity activities [23]. The instrument has been shown to have sufficient validity and reliability within a Norwegian population [24]. IPAQ data were reported following the recommendations outlined in the user manual [23].

### Sample Size

The sample size was calculated based on the primary outcome, assuming a clinically significant difference in relative VO_2peak_ between groups of 3.5 ml/kg/minute [25-27]. Using data from a previously conducted feasibility study [28], the SD was estimated to be 6 ml/kg/minute. With a power of 80% and a significance level of .05, the required sample size was determined to be 47 patients per group. To account for a 20% dropout rate at the 1-year follow-up, we aimed to include a total of 113 patients.

### Statistical Analysis

IBM SPSS Statistics (version 29) and Stata (version 18; StataCorp LLC) were used for statistical analysis. Continuous, normally distributed baseline data were analyzed using an independent *t* test to assess differences between groups, while the Pearson chi-square test was used for categorical data. Baseline differences between participants with and without 5-year outcome data were analyzed using the same statistical tests. Missing data were assessed according to established strategies for handling missing data in clinical trials [29]. Differences between groups in primary and secondary outcomes were evaluated using a mixed model for repeated measurements. This model included a random intercept of the patient, as well as group, follow-up time, the interaction between group and follow-up time, and the baseline outcome measure as fixed effects. The Mann-Whitney *U* test was used to analyze differences between groups in IPAQ data at the 5-year follow-up. Independent *t* tests and Pearson chi-square tests were used to assess differences between participants with and without 5-year primary outcome data. This analysis was conducted to determine whether imputation of missing values was necessary, in accordance with strategies for managing missing data in clinical trials [29]. All analyses were performed on an intention-to-treat basis, and all tests were 2-sided. Data are presented as mean (SD) unless otherwise specified. A *P* value <.05 was considered statistically significant.

### Ethical Considerations

The Regional Committee for Medical and Health Research Ethics (South-East, ID: 2016-1476) approved the study protocol, and the study was conducted in accordance with the Declaration of Helsinki. An updated study protocol for this follow-up study was submitted and approved. All patients provided written, informed consent before inclusion in the study. The study data reported in this manuscript are anonymous. The original and updated study protocols were registered on ClinicalTrials.gov with the IDs NCT03174106 and NCT05697120, respectively. The study protocol and 1-year results have been previously published [10,14].

## Results

**Figure 1:**
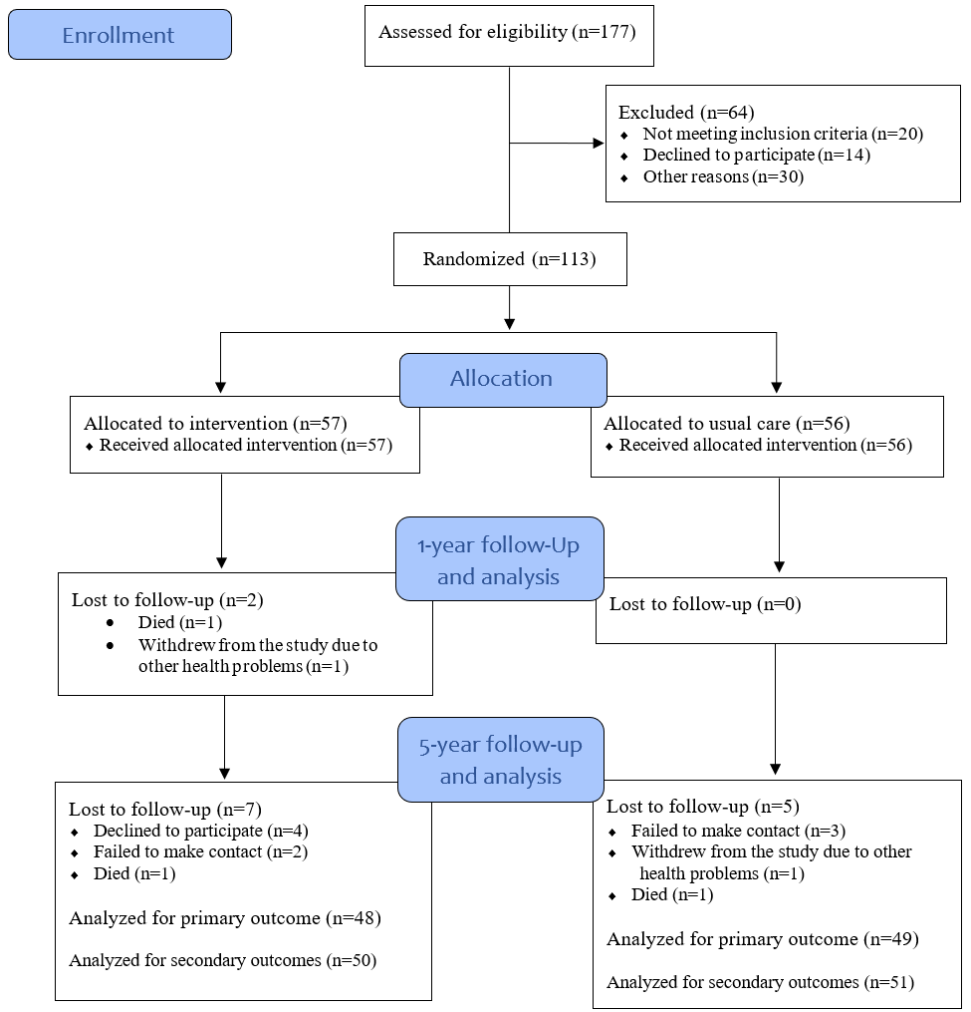
The CONSORT (Consolidated Standards of Reporting Trials) flow diagram.

A total of 177 patients were screened for eligibility at 2 CR centers between October 2017 and June 2018, of whom 113 were included and randomized to the IG or CG (see Figure 1). The 5-year follow-up was completed in September 2023, with a total of 101 patients assessed. In the IG, 2 patients were unable to perform the CPET due to advanced cancer and complex musculoskeletal disorders. In the CG, 2 patients were unable to perform the CPET due to complex musculoskeletal and mental health disorders. This left 97 patients (85.8%) available for analysis of the primary outcome and 101 patients (89.4%) for secondary outcomes (Figure 1).

There were no differences in baseline characteristics between participants with missing data at the 5-year follow-up and those without missing data. Missing data were assumed to be completely at random, as they were not related to any observed or unobserved variables [29]. Consequently, imputation of missing data was not performed [29].

No statistically significant differences in baseline characteristics were observed between the IG and CG (Table 1). Additionally, there were no differences between the groups in changes in medication from baseline to the 1-year follow-up [10] or at the 5-year follow-up.

At the 5-year follow-up, no statistically significant difference (*P*=.69) in VO_2peak_ was observed between the groups. Other outcomes evaluated at the 5-year follow-up are presented in Table 2. A statistically significant difference was found in exercise habits (*P*=.04), favoring the IG, and in triglycerides, *P*=.048), favoring the CG. No statistically significant differences were observed between the CG and IG for any other outcomes (Table 2).

The distribution of EQ-5D scores at baseline, 1-year follow-up, and 5-year follow-up is presented in Multimedia Appendix 1.

During the 1-year follow-up period, 11 patients experienced a new cardiac event (5 in the CG and 6 in the IG). Of these, 6 were cases of recurrent or new CAD (3 in the CG and 3 in the IG), and 4 were cases of AF (2 in the CG and 2 in the IG). Between the 1- and 5-year follow-ups, 21 patients experienced a new cardiac event, including 11 in the CG and 10 in the IG. Of these events, 9 were cases of recurrent or new CAD (8 in the CG and 1 in the IG), 5 were cases of AF (2 in the CG and 3 in the IG), 1 patient in the IG underwent valve surgery, and another survived a cardiac arrest (IG). Additionally, 2 patients in the IG underwent angiography, and 3 patients experienced other heart-related events (1 in the CG and 2 in the IG).

During the first year after CR, 49 patients experienced different kinds of diseases or health problems that affected their ability to adhere to a healthy behavior for 4 weeks or more. This included musculoskeletal disorders (n=34), pulmonary diseases (n=2), diseases in other organs (n=8), complex pain (n=2), and other disorders (n=3). The IG reported statistically significant (*P*<.001) more health problems compared with the CG the first year after CR. From the 1-year follow-up to the 5-year follow-up, 54 patients reported challenges with diseases or health problems, including cancer (n=6), serious COVID-19 (n=2), musculoskeletal disorders (n=30), psychological stress (n=11), and other conditions (n=5). There were no statistically significant differences (*P*=.32) between the IG and CG at a 5-year follow-up regarding diseases or health problems that impacted the ability to adhere to healthy behaviors.

At the 5-year follow-up, the CG reported walking significantly more (MET-minutes/week) than the IG, as assessed by the IPAQ (*P*=.03). There were no statistically significant differences between the groups in any other physical activity domains evaluated by the IPAQ (moderate MET-minutes/week, *P*=.69; vigorous MET-minutes/week, *P*=.20; and total MET-minutes/week, *P*=.64).

**Table 1:** Descriptive statistics of the sample at baseline and at 1- and 5-year follow-ups.

Variables		Total (n=113)	CG^a^ (n=56)	IG^b^ (n=57)	CG at 1 year	IG at 1 year	CG at 5 years	IG at 5 years
Age, mean (SD)		59.0 (8.7)	58.4 (8.2)	59.5 (9.1)	—^c^	—	—	—
Female, n (%)		25 (22.1)	16 (28.6)	9 (15.8)	—	—	—	—
Non-European, n (%)		4 (3.5)	—	4 (7.0)	—	—	—	—
Married or cohabitant, n (%)		89 (78.8)	43 (76.8)	46 (80.7)	—	—	—	—
Current smoker, n (%)		4 (3.5)	1 (1.8)	3 (5.3)	3 (5.4)	2 (3.5)	2 (3.6)	2 (3.5)
Body weight, mean (SD)		90.2 (16.9)	88.5 (17.0)	91.8 (16.8)	88.6 (17.6)	90.4 (16.6)	89.2 (17.6)	90.6 (18.2)
**Disease, n (%)**								
	Acute coronary syndrome	44 (38.9)	22 (39.3)	22 (38.6)	—	—	—	—
	Coronary artery disease	39 (34.5)	19 (33.9)	20 (35.1)	—	—	—	—
	Valve	19 (16.8)	8 (14.3)	11 (19.3)	—	—	—	—
	Cardiac arrest	1 (0.9)	1 (1.8)	—	—	—	—	—
	Atrial fibrillation	3 (2.7)	1 (1.8)	2 (3.5)	—	—	—	—
	Other	7 (6.2)	5 (8.9)	2 (3.5)	—	—	—	—
**Treatment, n (%)**								
	Percutaneous coronary intervention	55 (48.7)	26 (46.4)	29 (50.9)	—	—	—	—
	Coronary artery bypass graft	22 (19.5)	12 (21.4)	10 (17.5)	—	—	—	—
	Valve surgery	19 (16.8)	8 (14.3)	11 (19.3)	—	—	—	—
	Implantable cardioverter-defibrillator	2 (1.8)	1 (1.8)	1 (1.8)	—	—	—	—
	Pacemaker	2 (1.8)	2 (3.6)	—	—	—	—	—
	Conservatively	10 (8.8)	6 (10.7)	4 (7.0)	—	—	—	—
	Other	3 (2.7)	1 (1.8)	2 (3.5)	—	—	—	—
**Medication, n (%)**								
	Betablocker	69 (61.1)	32 (57.1)	37 (64.9)	—	—	22 (39.3)	23 (40.4)
	Statins	96 (85.0)	45 (80.4)	51 (89.5)	—	—	41 (73.2)	43 (75.4)
	Acetylsalicylic acid^d^ + plate inhibitor	75 (66.4)	39 (69.6)	36 (63.2)	—	—	36 (64.3)^d^	33 (57.9)^d^
	Antihypertensive	55 (48.7)	29 (51.8)	26 (45.6)	—	—	32 (57.1)	30 (52.6)
**Type of cardiac rehabilitation, n (%)**								
	1 week	35 (31.0)	17 (30.4)	18 (31.6)	—	—	—	—
	4 weeks	40 (35.4)	20 (35.7)	20 (35.1)	—	—	—	—
	12 weeks	38 (33.6)	19 (33.9)	19 (33.3)	—	—	—	—
**Physical activity (International Physical Activity Questionnair), median (range)**								
	Walking (MET^e^-minutes/week)	—	—	—	—	—	809 (0-4851)	429 (0-11,946)
	Moderate (MET-minutes/week)	—	—	—	—	—	480 (0-13,440)	720 (0-8640)
	Vigorous (MET-minutes/week)	—	—	—	—	—	400 (0-11,520)	720 (0-6720)
	Total (MET-minutes/week)	—	—	—	—	—	2553 (132-27,600)	2243 (0-11,946)
**Cardiopulmonary exercise test** **, mean (SD)**								
	Peak oxygen uptake (ml/kg/minute)	29.6 (7.7)	29.9 (6.7)	29.4 (8.7)	28.7 (6.9)	31.0 (8.6)	28.1 (6.7)	27.8 (8.5)
	Peak oxygen uptake (l/minute)	2.64 (0.74)	2.63 (0.75)	2.65 (0.74)	2.48 (0.68)	2.71 (0.73)	2.48 (0.72)	2.48 (0.73)
	Time to exhaustion (seconds)	601 (124)	603 (102)	598 (144)	632 (120)	671 (130)	592 (117)	579 (134)
	Peak incline (%)	10.9 (3.6)	11.0 (3.0)	10.8 (4.2)	11.8 (3.8)	13.0 (4.1)	10.1 (3.9)	9.8 (3.8)
	Peak velocity (km/hour)	6.8 (1.6)	6.8 (1.5)	6.8 (1.7)	6.8 (1.5)	7.0 (1.8)	6.7 (1.4)	6.8 (1.5)
	Maximal heart rate (beats/minute)	160 (20)	161 (20)	159 (19)	164 (20)	164 (17)	161 (18)	159 (21)
	Peak respiratory exchange ratio	1.16 (0.1)	1.16 (0.1)	1.15 (0.11)	1.20 (0.09)	1.20 (0.08)	1.20 (0.07)	1.20 (0.09)
	Rate of perceived exertion (Borg Scale, scored from 6 to 20)	17.8 (1.0)	17.8 (1.0)	17.7 (1.1)	17.5 (1.2)	17.8 (1.3)	17.6 (1.4)	17.6 (1.4)
**Blood pressure, mean (SD)**								
	Systolic (mmHg)	135 (17)	136 (18)	133 (17)	145 (20)	143 (19)	135 (18)	133 (18)
	Diastolic (mmHg)	81 (9)	81 (8)	81 (10)	84 (11)	86 (10)	80 (12)	80 (12)
**Lipid profile**								
	Low-density lipoprotein-cholesterol (mmol/l)	2.2 (0.9)	2.3 (0.9)	2.2 (0.8)	2.3 (0.9)	2.3 (1.0)	2.3 (1.4)	2.1 (0.8)
	High-density lipoprotein-cholesterol (mmol/l)	1.3 (0.4)	1.3 (0.4)	1.2 (0.4)	1.4 (0.5)	1.3 (0.4)	1.4 (0.4)	1.3 (0.4)
	Total cholesterol (mmol/l)	4.0 (1.0)	4.0 (1.0)	4.0 (0.9)	3.9 (1.0)	4.1 (1.0)	4.0 (1.5)	3.8 (0.8)
	Triglycerides (mmol/l)	1.4 (0.9)	1.3 (1.0)	1.6 (0.9)	1.2 (0.7)	1.6 (1.5)	1.2 (0.7)	1.5 (1.0)
Exercise habits, mean (SD)		1.4 (1.5)	1.3 (1.5)	1.6 (1.5)	1.9 (1.6)	3.0 (1.9)	1.7 (1.7)	2.6 (2.8)
**HeartQoL^f^, mean (SD)**								
	Physical	2.5 (0.6)	2.5 (0.5)	2.4 (0.6)	2.6 (0.5)	2.7 (0.5)	2.5 (0.5)	2.5 (0.5)
	Emotional	2.4 (0.6)	2.3 (0.7)	2.4 (0.6)	2.3 (0.7)	2.5 (0.7)	2.4 (0.6)	2.4 (0.6)
	Global	2.4 (0.5)	2.4 (0.5)	2.4 (0.5)	2.5 (0.5)	2.6(0.5)	2.5 (0.5)	2.5 (0.5)
EQ VAS^g^, mean (SD)		71 (16)	72 (14)	69 (18)	75 (12)	78 (16)	74 (13)	70 (19)

^a^CG: control group.

^b^IG: intervention group.

^c^Not applicable.

^d^At the 5-year follow-up, the number and percentage given are for ASA only.

^e^MET: metabolic equivalent.

^f^HeartQoL: disease-specific Health-Related Quality of Life Questionnaire (scored from 0 to 3), which comprises 3 domains (physical, emotional, and global quality of life).

^g^VAS: Visual Analog Scale.

**Table 2:** Differences between groups at 4 years after the intervention, adjusted for baseline values.

Outcomes				Mean difference^a^		95% CI		*P* value
**Cardiopulmonary exercise test** **(n=97)**								
		Peak oxygen uptake (ml/kg/minute)		0.29		–1.13 to 1.70		.69
		Peak oxygen uptake (l/minute)		–0.0		–0.12 to 0.12		.99
		Time to exhaustion (seconds)		–10.7		–46.6 to 25.1		.56
		Peak incline (%)		–0.4		–1.6 to 0.7		.46
		Peak velocity (km/hour)		0.2		–0.01 to 0.39		.06
		Body weight (kg) (n=101)		–1.23		–3.24 to 0.78		.23
**Blood pressure (n=97)**								
		Systolic (mmHg)		–1.5		–8.0 to 5.0		.65
		Diastolic (mmHg)		–0.3		–4.6 to 3.9		.89
**Lipid profile (n=101)**								
		Low-density lipoprotein-cholesterol (mmol/l)		0.024		–0.257 to 0.304		.87
		High-density lipoprotein-cholesterol (mmol/l)		–0.065		–0.169 to 0.039		.22
		Total cholesterol (mmol/l)		–0.039		–0.337 to 0.259		.80
		Triglycerides (mmol/l)		0.40		0.00 to 0.79		.048^b^
Exercise habits (n=101)				0.67		0.04 to 1.29		.04^b^
**HeartQoL^c^ (n=101)**								
	Physical		0.01		–0.15 to 0.17		.90	
	Emotional		–0.01		–0.21 to 0.19		.90	
	Global		0.01		–0.14 to 0.16		.92	
EQ Visual Analog Scale^d^ (n=101)				–2.49		–7.53 to 2.56		.33

^a^Mean difference is calculated as intervention – control.

^b^*P*<.05 between groups.

^c^HeartQoL: disease-specific Health-Related Quality of Life Questionnaire (scored from 0 to 3), which comprises 3 domains (physical, emotional, and global quality of life).

^d^The overall health status was measured using the Visual Analog Scale (0-100) in the EQ-5D questionnaire.

## Discussion

### Principal Findings

To the best of our knowledge, this is the first study to evaluate the long-term effects of individualized follow-up using an app during the first year after CR. Our primary finding indicates that the beneficial effects on VO_2peak_ and exercise performance observed 1 year after CR [10] had diminished 4 years after the intervention. For the primary outcome (VO_2peak_), we maintained adequate statistical power to detect a true difference between the groups at the 5-year follow-up. However, the results for secondary outcomes should be interpreted with caution.

Numerous factors may explain why the effects observed after 1 year of individualized follow-up [10] were no longer present at the 5-year follow-up, and we can only speculate whether these effects might have been sustained if the follow-up had continued. We believe the relationship between the supervisor and the patients in our study played a pivotal role in fostering the successful adherence to healthy behaviors observed 1 year after CR [10]. This was also highlighted by the patients in a qualitative study investigating their experiences with the follow-up provided [30]. The patients emphasized that the supervisor, the person behind the app, was the most significant factor in promoting adherence to healthy behavior [30]. Successful adherence in the context of cardiovascular risk reduction is often attributed to an ongoing, collaborative relationship between the health care provider and the patient [31]. The idea that close monitoring or follow-up can enhance adherence is widely recognized [1,3]; however, only a few studies have demonstrated this in real-world settings. In our study, follow-up was discontinued after 1 year, and the observed effects appeared to diminish over time. The app-based follow-up provided to the IG during the first year after CR in this study was grounded in the Transtheoretical Model, also known as the Stages of Change Model [32]. According to this model, health behavior change involves 6 stages: precontemplation, contemplation, preparation, action, maintenance, and termination. The model emphasizes that behavior change is a process that takes time and is not linear. For example, patients in the maintenance stage may quickly regress to the preparation stage after CR due to factors such as a new cardiac event or other diseases or health problems that challenge their ability to maintain healthy behaviors. The need for support varies across different stages and should be tailored to increase the likelihood of successful behavior change [32]. Our findings suggest that reaching the termination phase—where individuals are confident they will never revert to their previous unhealthy behaviors and require minimal support [32]—is unlikely to be achieved within a 1-year follow-up period. Whether it would be possible with extended follow-up beyond 1 year remains an open question.

Patients in the IG reported exercising significantly more than those in the CG at the 5-year follow-up. However, there were no statistically significant differences between the groups in variables that reflect the effects of exercise (eg, VO_2peak_ and exercise performance). Notably, the difference in exercise habits between the groups at 5 years was similar to that observed at the 1-year follow-up. A plausible explanation for the lack of differences in VO_2peak_ and exercise performance at the 5-year follow-up, despite the IG reporting higher levels of exercise, could be the lower intensity or shorter duration of exercise sessions after the individualized follow-up was discontinued. As expressed by the patients in the IG [30], the individualized follow-up during the first year after CR provided more structure and focus to their exercise sessions than they were able to achieve on their own. Based on IPAQ data, the CG and IG appear to be equally physically active at the 5-year follow-up, although the CG reported significantly higher levels of walking METs compared with the IG. The IG reported higher levels of physical activity at both moderate and vigorous intensities than the CG, which may correspond to the significant difference observed in exercise habits, although differences observed in moderate and vigorous intensities were not statistically significant. Additionally, there was a statistically significant difference in triglycerides at the 5-year follow-up, favoring the CG. However, the uncertainty regarding the clinical relevance of the observed difference renders the result insignificant, and, on average, both groups remained within normal ranges [33].

For other metabolic risk factors, there were no statistically significant differences between the groups. The mean difference in body weight between the IG and CG at the 5-year follow-up was >1 kg. Although this difference did not reach statistical significance, we believe it is noteworthy, as each kilogram of weight gain has been shown to increase the risk of type 2 diabetes mellitus by 7.3% [34]. This suggests there may be a clinically relevant difference in body weight favoring the IG. However, given a minimal clinically important difference in body weight of 1 kg and an SD of 17 kg (based on our sample), more than 4500 patients would be required in each group to achieve 80% statistical power at a 5% significance level. Another noteworthy finding, although not statistically significant, is the LDL-cholesterol levels. The mean LDL-cholesterol at the 5-year follow-up was 2.3 mmol/l in the CG and 2.1 mmol/l in the IG. Given that the sample includes patients both with and without CAD, these levels are not alarming. When examining LDL-cholesterol specifically for those with CAD or acute coronary syndrome as the referral cause, the mean LDL-cholesterol levels at 5 years were 1.70 (SD 0.62) mmol/l in the CG and 1.88 (SD 0.62) mmol/l in the IG. The elevated LDL-cholesterol levels observed appear to be a common finding in studies evaluating maintenance programs after CR [35-38]. A reasonable explanation for this trend is that these post-CR programs [35-38] primarily focus on exercise rather than specifically addressing metabolic risk factors such as LDL-cholesterol and body weight. When comparing our results regarding LDL-cholesterol with a cross-sectional study by Peersen and colleagues (n=1127) [4], our sample at the 5-year follow-up is slightly better than their sample, with a mean LDL-cholesterol of 2.02 mmol/l 2-36 months (median 16 months) after a cardiac event. However, it is important to note that treatment targets for LDL-cholesterol have changed from <1.8 mmol/l to <1.4 mmol/l [39] since the completion of the intervention in this study. This indicates that there remains a need to optimize lipid management after CR. For the most part, lipid management after CR is handled by patients’ general practitioners. For decades, studies on lipid profiles following a cardiac event or CR have shown that few patients meet the guidelines for secondary prevention. This raises the timely question of whether this responsibility should be prioritized more by general practitioners or shifted back to the specialist health service. Consistent with the 1-year follow-up results [10], there were no statistically significant differences between the groups in HRQL at the 5-year follow-up. Nevertheless, it is encouraging that HRQL in our sample remained at a high level. This is particularly important, as reduced HRQL has been associated with an increased incidence of depression and anxiety [40,41], both of which are well-established psychosocial risk factors for poor prognosis in patients with cardiovascular disease [2]. Compared with the reference values for HeartQoL from the EUROASPIRE IV survey (EUROpean Action on Secondary and Primary prevention through Intervention to Reduce Events), our sample demonstrated higher scores in the global domain as well as the physical and emotional HRQL domains at both follow-up points [18].

### Strengths and Limitations

A key strength of our study is the low dropout rate at both the 1- and 5-year follow-ups. Additionally, patients were recruited from 3 different CR programs, and the use of broad inclusion criteria further strengthens the generalizability of our findings. Our goal was to make the results applicable to the wider population of cardiac patients participating in CR. Given that CR programs vary widely both across and within countries, we included patients from 3 distinct CR programs delivered in different settings (residential and outpatient) and with varying durations (1, 4, and 12 weeks). Because of the uncertainty regarding whether adherence to healthy behaviors after CR is influenced by the type of CR program, randomization was stratified based on the specific CR program. By including all medically stable patients with cardiac diseases, we were able to capture a representative sample of a typical CR population. However, this broad inclusion criterion also posed challenges in reporting some outcomes, particularly metabolic risk factors. For example, patients treated for valve disease may not necessarily be advised to reduce LDL-cholesterol levels or body weight. To address this, descriptive statistics on subgroups and the clinical relevance of these outcomes have been provided. We believe that different subgroups of patients completing CR may derive varying benefits from the intervention. To explore this further, future studies could consider narrowing the inclusion criteria or significantly increasing the sample size to enable systematic subgroup analyses.

### Conclusion and Implications

This study demonstrates that the beneficial and clinically important effects of a 1-year individualized follow-up via an app post-CR [10] had ceased by 4 years after the intervention. Despite the growing emphasis on addressing challenges related to adherence to healthy behavior and the use of technology in international research strategies [6,42,43], as well as in key public policies, we believe that significant work remains before mHealth can be fully implemented in this context. However, our findings on feasibility [28], 1-year follow-up outcomes [10], patients’ experiences [30], and the results presented in this study can provide valuable guidance for developing an implementation strategy for mHealth interventions aimed at promoting adherence to healthy behaviors in the post-CR setting. Before developing such a strategy, it is essential to conduct a comprehensive investigation into the potential barriers and facilitators associated with its implementation, involving all relevant stakeholders. To fully understand the multifaceted challenges of adherence to healthy behavior after CR, this investigation must include input from policy makers, CR program managers, health care professionals, and, most importantly, patients—both those struggling with adherence and those who have successfully maintained adherence to secondary prevention recommendations over the years.

## Appendix

Multimedia Appendix 1 Distribution in percentage of reporting levels 1-5 by dimension, group, and measurement time point.

Multimedia Appendix 2 CONSORT-eHEALTH checklist (V 1.6.1).

## Abbreviations

CAD: coronary artery disease
CG: control group
CONSORT: Consolidated Standards of Reporting Trials
CPET: cardiopulmonary exercise test
CR: cardiac rehabilitation
HDL: high-density lipoprotein
HRQL: health-related quality of life
IG: intervention group
IPAQ: International Physical Activity Questionnaire
LDL: low-density lipoprotein
mHealth: mobile health
VAS: Visual Analog Scale
VO2max: maximal oxygen consumption
VO_2peak_: peak oxygen uptake

## Data Availability

The data sets generated and analyzed during this study are not publicly available due to the consent provided by the participants. However, they may be made available from the corresponding author upon reasonable request.

## References

[1] Ambrosetti M, Abreu A, Corrà Ugo, Davos CH, Hansen D, Frederix I, Iliou MC, Pedretti RF, Schmid J, Vigorito C, Voller H, Wilhelm M, Piepoli MF, Bjarnason-Wehrens B, Berger T, Cohen-Solal A, Cornelissen V, Dendale P, Doehner W, Gaita D, Gevaert AB, Kemps H, Kraenkel N, Laukkanen J, Mendes M, Niebauer J, Simonenko M, Zwisler AO (2021). Secondary prevention through comprehensive cardiovascular rehabilitation: from knowledge to implementation. 2020 update. A position paper from the Secondary Prevention and Rehabilitation Section of the European Association of Preventive Cardiology. Eur J Prev Cardiol.

[2] Piepoli MF, Hoes AW, Agewall S, Albus C, Brotons C, Catapano AL, Cooney M, Corrà Ugo, Cosyns B, Deaton C, Graham I, Hall MS, Hobbs FDR, Løchen Maja-Lisa, Löllgen Herbert, Marques-Vidal P, Perk J, Prescott E, Redon J, Richter DJ, Sattar N, Smulders Y, Tiberi M, Bart van der Worp H, van Dis I, Verschuren WMM, Authors/Task Force Members: (2016). Atherosclerosis.

[3] Pedretti RFE, Hansen D, Ambrosetti M, Back M, Berger T, Ferreira MC, Cornelissen V, Davos CH, Doehner W, de Pablo Y Zarzosa C, Frederix I, Greco A, Kurpas D, Michal M, Osto E, Pedersen SS, Salvador RE, Simonenko M, Steca P, Thompson DR, Wilhelm M, Abreu A (2023). How to optimize the adherence to a guideline-directed medical therapy in the secondary prevention of cardiovascular diseases: a clinical consensus statement from the European Association of Preventive Cardiology. Eur J Prev Cardiol.

[4] Peersen K, Munkhaugen J, Gullestad L, Liodden T, Moum T, Dammen T, Perk J, Otterstad JE (2017). The role of cardiac rehabilitation in secondary prevention after coronary events. Eur J Prev Cardiol.

[5] Kotseva K, Wood D, De Bacquer D, De Backer G, Rydén Lars, Jennings C, Gyberg V, Amouyel P, Bruthans J, Castro Conde A, Cífková Renata, Deckers JW, De Sutter J, Dilic M, Dolzhenko M, Erglis A, Fras Z, Gaita D, Gotcheva N, Goudevenos J, Heuschmann P, Laucevicius A, Lehto S, Lovic D, Miličić D, Moore D, Nicolaides E, Oganov R, Pajak A, Pogosova N, Reiner Z, Stagmo M, Störk Stefan, Tokgözoğlu Lale, Vulic D, EUROASPIRE Investigators (2016). EUROASPIRE IV: A European Society of Cardiology survey on the lifestyle, risk factor and therapeutic management of coronary patients from 24 European countries. Eur J Prev Cardiol.

[6] World Health Organization (WHO) (2011). mHealth: New Horizons for Health Through Mobile Technologies: Second Global Survey on eHealth.

[7] Beatty AL, Fukuoka Y, Whooley MA (2013). Using mobile technology for cardiac rehabilitation: a review and framework for development and evaluation. J Am Heart Assoc.

[8] Duscha BD, Piner LW, Patel MP, Craig KP, Brady M, McGarrah RW, Chen C, Kraus WE (2018). Effects of a 12-week mHealth program on peak VO and physical activity patterns after completing cardiac rehabilitation: a randomized controlled trial. Am Heart J.

[9] Snoek JA, Meindersma EP, Prins LF, Van't Hof AW, de Boer M, Hopman MT, Eijsvogels TM, de Kluiver EP (2021). The sustained effects of extending cardiac rehabilitation with a six-month telemonitoring and telecoaching programme on fitness, quality of life, cardiovascular risk factors and care utilisation in CAD patients: the TeleCaRe study. J Telemed Telecare.

[10] Lunde P, Bye A, Bergland A, Grimsmo J, Jarstad E, Nilsson BB (2020). Long-term follow-up with a smartphone application improves exercise capacity post cardiac rehabilitation: a randomized controlled trial. Eur J Prev Cardiol.

[11] Schulz KF, Altman DG, Moher D (2010). CONSORT 2010 Statement: updated guidelines for reporting parallel group randomised trials. BMC Med.

[12] Nilsson BB, Lunde P, Holm I (2019). Implementation and evaluation of the Norwegian Ullevaal model as a cardiac rehabilitation model in primary care. Disabil Rehabil.

[13] Bergum H, Sandven I, Abdelnoor M, Anderssen SA, Grimsmo J, Rivrud DE, Myhr NE, Vold MB, Stenbakken C, Lidfors B, Dufseth L, Klemsdal TO (2022). Randomized trial of cardiovascular prevention in Norway combining an in-hospital lifestyle course with primary care follow-up: the Hjerteløftet study. Eur J Prev Cardiol.

[14] Lunde P, Bye A, Bergland A, Nilsson BB (2019). Effects of individualized follow-up with a smartphone-application after cardiac rehabilitation: protocol of a randomized controlled trial. BMC Sports Sci Med Rehabil.

[15] Borg GA (1982). Psychophysical bases of perceived exertion. Med Sci Sports Exerc.

[16] Oldridge N, Höfer S, McGee H, Conroy R, Doyle F, Saner H, (for the HeartQoL Project Investigators) (2014). The HeartQoL: Part I. Development of a new core health-related quality of life questionnaire for patients with ischemic heart disease. Eur J Prev Cardiol.

[17] Oldridge N, Höfer S, McGee H, Conroy R, Doyle F, Saner H, (for THPI (2014). The HeartQoL: part II. Validation of a new core health-related quality of life questionnaire for patients with ischemic heart disease. Eur J Prev Cardiol.

[18] De Smedt D, Clays E, Höfer S, Oldridge N, Kotseva K, Maggioni AP, Pogosova N, Dolzhenko M, De Bacquer D (2016). The use of HeartQoL in patients with coronary heart disease: association with risk factors and European reference values. The EUROASPIRE IV study of the European Society of Cardiology. Eur J Prev Cardiol.

[19] Kristensen MS, Zwisler A, Berg SK, Zangger G, Grønset CN, Risom SS, Pedersen SS, Oldridge N, Thygesen LC (2016). Validating the HeartQoL questionnaire in patients with atrial fibrillation. Eur J Prev Cardiol.

[20] Zangger G, Zwisler A, Kikkenborg Berg S, Kristensen MS, Grønset CN, Uddin J, Pedersen SS, Oldridge NB, Thygesen LC (2018). Psychometric properties of HeartQoL, a core heart disease-specific health-related quality of life questionnaire, in Danish implantable cardioverter defibrillator recipients. Eur J Prev Cardiol.

[21] Grønset CN, Thygesen LC, Berg SK, Zangger G, Kristensen MS, Sibilitz KL, Pedersen SS, Oldridge NB, Zwisler A (2019). Measuring HRQoL following heart valve surgery: the HeartQoL questionnaire is a valid and reliable core heart disease instrument. Qual Life Res.

[22] Herdman M, Gudex C, Lloyd A, Janssen M, Kind P, Parkin D, Bonsel G, Badia X (2011). Development and preliminary testing of the new five-level version of EQ-5D (EQ-5D-5L). Qual Life Res.

[23] Craig CL, Marshall AL, Sjöström M, Bauman AE, Booth ML, Ainsworth BE, Pratt M, Ekelund U, Yngve A, Sallis JF, Oja P (2003). International physical activity questionnaire: 12-country reliability and validity. Med Sci Sports Exerc.

[24] Kurtze N, Rangul V, Hustvedt B (2008). Reliability and validity of the international physical activity questionnaire in the Nord-Trøndelag health study (HUNT) population of men. BMC Med Res Methodol.

[25] Gulati M, Pandey DK, Arnsdorf MF, Lauderdale DS, Thisted RA, Wicklund RH, Al-Hani AJ, Black HR (2003). Exercise capacity and the risk of death in women: the St James Women Take Heart Project. Circulation.

[26] Myers J, Prakash M, Froelicher V, Do D, Partington S, Atwood JE (2002). Exercise capacity and mortality among men referred for exercise testing. N Engl J Med.

[27] Myers J, McAuley P, Lavie CJ, Despres J, Arena R, Kokkinos P (2015). Physical activity and cardiorespiratory fitness as major markers of cardiovascular risk: their independent and interwoven importance to health status. Prog Cardiovasc Dis.

[28] Lunde P, Nilsson BB, Bergland A, Bye A (2019). Feasibility of a mobile phone app to promote adherence to a heart-healthy lifestyle: single-arm study. JMIR Form Res.

[29] Dziura JD, Post LA, Zhao Q, Fu Z, Peduzzi P (2013). Strategies for dealing with missing data in clinical trials: from design to analysis. Yale J Biol Med.

[30] Lunde P, Bye A, Bruusgaard KA, Hellem E, Nilsson BB (2022). Patients' experiences of using a smartphone app after cardiac rehabilitation: qualitative study. JMIR Hum Factors.

[31] Cohen SM (2009). Concept analysis of adherence in the context of cardiovascular risk reduction. Nurs Forum.

[32] Prochaska J.O, Redding A.C, Evers K.E, Glanz K, Rimer BK, Viswanath K (2015). The transtheoretical model and stages of change. Health Behavior: Theory, Research, and Practice.

[33] Yang G, Mason AM, Wood AM, Schooling CM, Burgess S (2024). Dose-response associations of lipid traits with coronary artery disease and mortality. JAMA Netw Open.

[34] Koh-Banerjee P, Wang Y, Hu FB, Spiegelman D, Willett WC, Rimm EB (2004). Changes in body weight and body fat distribution as risk factors for clinical diabetes in US men. Am J Epidemiol.

[35] Avila A, Claes J, Buys R, Azzawi M, Vanhees L, Cornelissen V (2020). Home-based exercise with telemonitoring guidance in patients with coronary artery disease: does it improve long-term physical fitness?. Eur J Prev Cardiol.

[36] Janssen V, De Gucht Veronique, van Exel Henk, Maes S (2013). Beyond resolutions? A randomized controlled trial of a self-regulation lifestyle programme for post-cardiac rehabilitation patients. Eur J Prev Cardiol.

[37] Skobel E, Knackstedt C, Martinez-Romero A, Salvi D, Vera-Munoz C, Napp A, Luprano J, Bover R, Glöggler S, Bjarnason-Wehrens B, Marx N, Rigby A, Cleland J (2017). Internet-based training of coronary artery patients: the Heart Cycle Trial. Heart Vessels.

[38] Madssen E, Arbo I, Granøien Ingrid, Walderhaug L, Moholdt T (2014). Peak oxygen uptake after cardiac rehabilitation: a randomized controlled trial of a 12-month maintenance program versus usual care. PLoS One.

[39] Mach F, Baigent C, Catapano AL, Koskinas KC, Casula M, Badimon L, Chapman MJ, De Backer GG, Delgado V, Ference BA, Graham IM, Halliday A, Landmesser U, Mihaylova B, Pedersen TR, Riccardi G, Richter DJ, Sabatine MS, Taskinen M, Tokgozoglu L, Wiklund O, ESC Scientific Document Group (2020). 2019 ESC/EAS Guidelines for the management of dyslipidaemias: lipid modification to reduce cardiovascular risk. Eur Heart J.

[40] Mayou RA, Gill D, Thompson DR, Day A, Hicks N, Volmink J, Neil A (2000). Depression and anxiety as predictors of outcome after myocardial infarction. Psychosom Med.

[41] Dickens C, Cherrington A, McGowan L (2012). Depression and health-related quality of life in people with coronary heart disease: a systematic review. Eur J Cardiovasc Nurs.

[42] World Health Organization (WHO) (2003). Adherence to Long-Term Therapies: Evidence for Action.

[43] World Health Organization (2013). WHO Global NCD Action Plan 2013-2020.

