# Supplementary material for: Effects of Individualized Follow-Up With an App Postcardiac Rehabilitation: Five-Year Follow-Up of a Randomized Controlled Trial
Source: J Med Internet Res. 2025 Feb 13;27:e60256. doi: 10.2196/60256 (PMC11888047; doi:10.2196/60256)
Supplement: Multimedia Appendix 1 [file jmir_v27i1e60256_app1.docx]

| **Tabel S1.** Distribution in percentage of reporting level 1 to 5 by dimension, group and measure time point | | | | | | |
| --- | --- | --- | --- | --- | --- | --- |
| **EQ-5D DIMENSION** | Control group | | | Intervention group | | |
|  | Baseline  (n=56) | 1-year  (n=56) | 5-year  (n=51) | Baseline  (n=57) | 1-year  (n=55) | 5-year  (n=50) |
| Level 1  Level 2  MOBILITY Level 3  Level 4  Level 5 | 85.7%  8.9%  5.4%  0%  0% | 89.3%  5.4%  3.6%  1.8%  0% | 80.4%  13.7%  3.9%  2%  0% | 86%  12.3%  0%  1.8%  0% | 96.4%  3.6%  0%  0%  0% | 70%  28%  0%  0%  2% |
| Level 1  Level 2  SELF-CARE Level 3  Level 4  Level 5 | 92.9%  7.1%  0%  0%  0% | 98.2%  1.8%  0%  0%  0% | 94.1%  3.9%  2%  0%  0% | 98.2%  1.8%  0%  0%  0% | 98.2%  1.8%  0%  0%  0% | 96%  4%  0%  0%  0% |
| Level 1  Level 2  USUAL ACTIVITY Level 3  Level 4  Level 5 | 80.4%  16.1%  3.6%  0%  0% | 85.7%  12.5%  1.8%  0%  0% | 84.3%  13.7%  2%  0%  0% | 68.4%  26.3%  5.3%  0%  0% | 80%  20%  0%  0%  0% | 72%  24%  4%  0%  0% |
| Level 1  Level 2  PAIN / Level 3  DISCOMFORT Level 4  Level 5 | 42.9%  44.6%  7.1%  3.6%  1.8% | 46.4%  32.1%  12.5%  8.9%  0% | 37.3%  37.3%  15.7%  9.8%  0% | 38.6%  50.9%  8.8%  1.8%  0% | 54.5%  30.9%  12.7%  1.8%  0% | 38%  50%  10%  2%  0% |
| Level 1  Level 2  ANXIETY / Level 3  DEPRESSION Level 4  Level 5 | 55.4%  42.9%  1.8%  0%  0% | 60.7%  33.9%  1.8%  3.6%  0% | 66.7%  29.4%  2%  2%  0% | 64.9%  29.8%  5.3%  0%  0% | 69.1%  23.6%  7.3%  0%  0% | 60%  32%  8%  0%  0% |
